# The anti-microbial peptide rCRAMP is strongly upregulated during experimental arthritis in the rat

**DOI:** 10.1186/1479-5876-9-S2-O7

**Published:** 2011-11-23

**Authors:** Markus H Hoffmann, Martin Herrmann, Birgitta Agerberth, Rikard Holmdahl

**Affiliations:** 1 Dept. of Medical Biochemistry and Biophysics, Karolinska Institutet, Stockholm, Sweden; 2 Dept. of Internal Medicine 3, University of Erlangen-Nurnberg, Erlangen, Germany

## Background

Rheumatoid arthritis (RA) is characterized by an inflammatory and destructive infiltration of various immune cells into the synovium of the joint. The first cells to arrive at the site of inflammation and the main cell type in synovial fluid are neutrophil granulocytes that locally release cytotoxic agents, antimicrobial peptides, proteases, and other inflammatory mediators, thus fuelling inflammation and damaging tissue. The aim of this project is to elucidate if locally released anti-microbial peptides contribute to the pathogenic events of arthritis.

## Materials and methods

Expression of the rat cathelicidin related anti-microbial peptide (rCRAMP) during the course of pristane-induced arthritis (PIA) in DA rats was determined in blood, synovial blood, joint extracts, lymph nodes, spleens and livers by Western blotting and FACS. Formation of neutrophil extracellular traps (NET) was analyzed by DAPI staining. IFN-alpha was determined in sera by ELISA.

## Results

In PIA, an animal model of arthritis that closely mimics RA, rCRAMP is strongly upregulated in the joints of arthritic animals. Interestingly, the strongest overexpression is seen around onset of clinical arthritis. rCRAMP expression is also upregulated in the blood of pristane-injected rats suffering even before the onset of clinical arthritis. Upregulation after pristane injection can as well be seen in spleen, but not in lymph nodes or the liver. rCRAMP is mainly expressed in His48+ CD62L- granulocytes and a smaller fraction of monocytes. Pristane application induces NETs and causes accumulation of a low density neutrophils that copurify with PBMC. The increased expression of rCRAMP coincides with elevated serum levels of type I Interferons in PIA rats.

## Discussion

Our results show strong upregulation of rCRAMP coinciding with pathological events in PIA. This might be due to at connection between rCRAMP forming complexes with nucleic acids that are locally released from damaged cells and activate of Interferon-producing cells.

